# Supplementary material for: Improvement of mRNA Delivery Efficiency to a T Cell Line by Modulating PEG-Lipid Content and Phospholipid Components of Lipid Nanoparticles
Source: Pharmaceutics. 2021 Dec 6;13(12):2097. doi: 10.3390/pharmaceutics13122097 (PMC8706876; doi:10.3390/pharmaceutics13122097)
Supplement: Supplementary file 1 [file pharmaceutics-13-02097-s001.zip › pharmaceutics-1458905-supplementary.pdf]

pharmaceutics

Supplementary Materials: Improvement of mRNA Delivery Ef-Ficiency to a T Cell Line by Modulating PEG-Lipid Content and Phospholipid Components of Lipid Nanoparticles

Hiroki Tanaka, Ryo Miyama, Yu Sakurai, Shinya Tamagawa, Yuta Nakai, Kota Tange, Hiroki Yoshioka and Hide-taka Akita

1. Supplementary tables

TableS1. Detailed list of supplier's information of the reagents

TableS2. The stock solution list of the lipids

TableS3. The composition of lipid solution for each LNP_{ssPalm}

TableS4. The data of hemolysis assay for Figure 5

TableS5. The data of hemolysis assay for Figure S3

TableS6. The data of hemolysis assay for Figure S5

2. Supplementary figures

Figure S1. *In vitro* toxicity evaluation of the LNP_{ssPalm}

Figure S2. Homogeneous expression of mRNA by LNP_{ssPalm} and EP

Figure S3. Effects of PEG-lipid amount on hemolysis activity

Figure S4. Apparent pKa of the LNPs with different amount of PEG-lipid

Figure S5. Effects of phospholipids on hemolysis activity

Figure S6. Transfection activity against other cell lines

Figure S7. Transfection activity in the presence of the CME inhibitor

Figure S8. Schematic illustration of integrated stress responses (ISRs)

3. References for Supporting Information

Table S1. Detailed list of supplier's information of the reagents.

Reagent	Size	Manufacturer	Product Number
ssPalmO-Phe-P4C2	1 g	NOF CORPORATION	COATSOME® SS-OP
Ethanol 99.5%	500 mL	Nacalai tesque	14712-05
SUNBRIGHT® GM-020(DMG-PEG)	1 g	NOF CORPORATION	GM-020
DiD	5 mg	Biotium	60014
COATSOME® ME-6081 (POPE)	1 g	NOF CORPORATION	ME-6081
COATSOME® MC-8181 (DOPC)	1 g	NOF CORPORATION	MC-8181
COATSOME® MC-6081 (POPC)	1 g	NOF CORPORATION	MC-6081
Cholesterol Sigma Grade, ≥ 99%	5 g	SIGMA Aldrich	C8667-5G
MES	100 g	Nacalai tesque	02442-44
DL-Malic Acid	500 g	Nacalai tesque	21029-55

Sodium Chloride	500 g	Nacalai tesque	31320-05
HEPES	500 g	DOJINDO LABORATORIES	342-01375
Sodium Hydroxide	500 g	Nacalai tesque	06338-75
UltraPure™ DNase/RNase-Free Distilled Water	500 mL	Invitrogen™	10977023
D-PBS(-) without Ca and Mg, liquid	500 mL	Nacalai tesque	14249-24
Quant-iT™ RiboGreen® RNA reagent	1 mL	Invitrogen™	R11491
Polyoxyethylene(10) Octylphenyl Ether	500 mL	FUJIFILM Wako Pure Chemical Corporation	168-11805
Opti-MEM™ I Reduced Serum Medium, no phenol red	500 mL	Gibco™	11058021
CleanCap Fluc mRNA	1 mg	TriLink BioTechnologies	L-7602-1000
CleanCap® EGFP mRNA (5moU)	100 µg	TriLink BioTechnologies	L-7201-100
Lipofectamine™ MessengerMAX™ Transfection Reagent	0.3 mL	Invitrogen™	LMRNA003
D-Luciferin Potassium Salt	1 g	FUJIFILM Wako Pure Chemical Corporation	126-05116
RPMI-1640 Medium, With L-glutamine and sodium bicarbonate	500 mL	SIGMA Aldrich	R8758
100mM-Sodium Pyruvate Solution(100x)	100 mL	Nacalai tesque	06977-34
45w/v% D(+)-Glucose Solution	100 mL	FUJIFILM Wako Pure Chemical Corporation	079-05511
1mol/l-HEPES Buffer Solution	100 mL	Nacalai tesque	17557-94
Penicillin-Streptomycin Mixed Solution(Stabilized)	100 mL	Nacalai tesque	26253-84
Rabbit monoclonal [E90] to EIF2S1 (phospho S51)	100 µL	Abcam	ab32157
Donkey anti-Rabbit IgG (H+L) Highly Cross-Adsorbed Secondary Antibody, Alexa Fluor 647	1 mg	Invitrogen™	A-31573
Paraformaldehyde	100 g	FUJIFILM Wako Pure Chemical Corporation	160-16061
Methanol	1 L	FUJIFILM Wako Pure Chemical Corporation	138-14521
Sodium Azide	500 g	Wako Pure Chemical Industries, Ltd.	194-01275
Albumin, Bovine Serum, General Grade, pH7.0	100 g	Nacalai tesque	01860-07

Dulbecco PBS (-) powder "Nissui"	100 g	Nissui Pharmaceutical Co., Ltd.	05913
Dimethyl Sulfoxide	50 mL	FUJIFILM Wako Pure Chemical Corporation	041-29351
Pitstop® 2, Novel cell-permeable clathrin inhibitor	5 mg	Abcam	ab120687
Dynole® 34-2, dynamin I and dynamin II inhibitor	5 mg	Abcam	ab120463
InSolution™ Cytochalasin D	1 mg	Sigma-Aldrich	504776
Genistein	100 mg	Santa Cruz Biotechnology	sc-3515
siClatrin	10 μM	Santa Cruz Biotechnology	sc-35067
Heparin Sodium Injection 5,000 units/5mL MOCHIDA	5 mL	MOCHIDA PHARMACEUTICAL CO.,LTD.	224122458
Cell Counting Kit-8	2500 tests	DOJINDO LABORATORIES	343-07623
TNS(6-(p-Toluidino)-2-naphthalenesulfonic acid sodium salt)	250 mg	Sigma-Aldrich	T9792-250MG
Sodium Dihydrogenphosphate Dihydrate	500 g	FUJIFILM Wako Pure Chemical Corporation	192-02815
Tris(hydroxymethyl)aminomethane	500 g	Nacalai tesque	35406-75
Steady-Glo® Luciferase Assay System	100 mL	Promega	E2520

Table S2. The stock solution list of the lipids.

Lipid	MW	Concentration	Storage Condition	Solvent
SS-OP	1173.8	10 mM	−20℃	EtOH
DOPC	786.1	5 mM	−20℃	EtOH
POPE	718.0	5 mM	−20℃	EtOH
Cholesterol	386.7	10 mM	−20℃	EtOH
DMG-PEG2000	2521.0	1 mM	−20℃	EtOH

Table S3. The composition of the lipid solution for each LNP_{ssPalm}

		SSOP	DOPC	POPE	Cholesterol	PEG	EtOH
SSOP/DOPC/Cholesterol/PEG = 52.5/7.5/40/0.375	nmol	420	60		320	3	
	μL	42	12		32	3	111
SSOP/DOPC/Cholesterol/PEG = 52.5/7.5/40/0.75	nmol	420	60		320	6	
	μL	42	12		32	6	108
SSOP/DOPC/Cholesterol/PEG = 52.5/7.5/40/1.5	nmol	420	60		320	12	
	μL	42	12		32	12	102
SSOP/DOPC/Cholesterol/PEG = 52.5/7.5/40/3	nmol	420	60		320	24	
	μL	42	12		32	24	90
SSOP/POPE/Cholesterol/PEG = 50/15/35/0.375	nmol	400		120	280	3	
	μL	40		24	28	3	105
SSOP/POPE/Cholesterol/PEG = 50/15/35/0.75	nmol	400		120	280	6	
	μL	40		24	28	6	102
SSOP/POPE/Cholesterol/PEG = 50/15/35/1.5	nmol	400		120	280	12	
	μL	40		24	28	12	96
SSOP/POPE/Cholesterol/PEG = 50/15/35/3	nmol	400		120	280	24	
	μL	40		24	28	24	84

Table S4. The data for the hemolysis assay for Figure 5.

pH	5.5	5.7	5.9	6.1	6.3	6.5
SSOP/POPE/Cholesterol/PEG = 50/15/35/0.75	1.025 ± 0.013	1.012 ± 0.026	0.812 ± 0.023	0.501 ± 0.025	0.275 ± 0.034	0.159 ± 0.034
SSOP/POPE/Cholesterol/PEG = 50/15/35/1.5	1.020 ± 0.024	1.014 ± 0.003	0.688 ± 0.093	0.398 ± 0.014	0.211 ± 0.014	0.126 ± 0.022
Negative control (PBS instead of LNP)	0.092 ± 0.007	0.076 ± 0.005	0.076 ± 0.005	0.069 ± 0.006	0.070 ± 0.007	0.068 ± 0.005
Positive control (Triton X100 instead of LNP)	0.857 ± 0.013	0.916 ± 0.026	0.937 ± 0.015	0.970 ± 0.065	0.950 ± 0.011	0.958 ± 0.014

Absorbance (mean with SD) was shown.

Table S5. The data for the hemolysis assay for Figure S3.

Lipid concentration	12.5 μM			25 μM		
DMG-PEG2000 modification	0.75%	1.5%	3%	0.75%	1.5%	3%
pH5.5	0.272 ± 0.112	0.425 ± 0.047	0.410 ± 0.051	0.458 ± 0.219	0.685 ± 0.076	0.608 ± 0.045
pH6.5	0.088 ± 0.009	0.091 ± 0.004	0.089 ± 0.002	0.092 ± 0.004	0.091 ± 0.004	0.084 ± 0.007
pH7.4	0.084 ± 0.009	0.083 ± 0.004	0.083 ± 0.002	0.091 ± 0.007	0.077 ± 0.006	0.079 ± 0.009
Lipid concentration	50 μM			100 μM		
DMG-PEG2000 modification	0.75%	1.5%	3%	0.75%	1.5%	3%
pH5.5	0.743 ± 0.293	1.018 ± 0.076	0.883 ± 0.037	0.925 ± 0.129	1.053 ± 0.055	1.000 ± 0.076
pH6.5	0.111 ± 0.021	0.098 ± 0.006	0.085 ± 0.006	0.143 ± 0.037	0.137 ± 0.008	0.098 ± 0.008
pH7.4	0.091 ± 0.007	0.079 ± 0.002	0.076 ± 0.006	0.102 ± 0.01	0.084 ± 0.005	0.082 ± 0.006
Lipid concentration	200 μM					
DMG-PEG2000 modification	0.75%	1.5%	3%	Positive control (Triton X100 instead of LNP)	Negative control (PBS instead of LNP)	
pH5.5	1.061 ± 0.04	1.113 ± 0.079	1.177 ± 0.014	0.987 ± 0.052	0.120 ± 0.015	
pH6.5	0.250 ± 0.107	0.207 ± 0.005	0.136 ± 0.033	1.081 ± 0.042	0.065 ± 0.005	
pH7.4	0.141 ± 0.033	0.107 ± 0.011	0.090 ± 0.004	1.090 ± 0.042	0.067 ± 0.004	

The composition was ssPalm/POPE/Cholesterol = 50/15/35. Absorbance (mean with SD) was shown.

Table S6. The data for the hemolysis assay for Figure S5.

pH	5.5	5.7	5.9	6.1	6.3	6.5
SSOP/POPC/Cholesterol/PEG = 50/15/35/0.75	1.052 ± 0.029	1.048 ± 0.014	0.763 ± 0.036	0.399 ± 0.01	0.187 ± 0.035	0.138 ± 0.014
SSOP/POPE/Cholesterol/PEG = 50/15/35/0.75	1.025 ± 0.013	1.012 ± 0.026	0.812 ± 0.023	0.501 ± 0.025	0.275 ± 0.034	0.159 ± 0.034
Negative control (PBS instead of LNP)	0.092 ± 0.007	0.076 ± 0.005	0.076 ± 0.005	0.069 ± 0.006	0.07 ± 0.007	0.068 ± 0.005
Positive control (Triton X100 instead of LNP)	0.857 ± 0.013	0.916 ± 0.026	0.937 ± 0.015	0.97 ± 0.065	0.95 ± 0.011	0.958 ± 0.014

Absorbance (mean with SD) was shown.

Cellular toxicity is one of the important properties of LNPs. The toxicity of the LNP_{ssPalm} was evaluated by a cell counting kit-8 (DOJINDO LABORATORIES, Tokyo, Japan). Jurkat cells were seeded at 5×10^5 cells/mL to 96 well plate. The LNP_{ssPalm} with or without encapsulated luciferase IVT-mRNA was added to the well. Final concentrations of the total lipids (total of ssPalm, phospholipid, and cholesterol) were in the range from 3.125 μM to 1600 μM. As a control, mRNA-free LNP_{ssPalm} containing ccPalmO-Phe, a non-cleavable counterpart of the ssPalmO-Phe-P4C2 was used. The material showed toxicity against HeLa cells and HepG2 cells [1]. At 24 hours after the addition, the viability of the cells was evaluated according to the manufacture's protocol.

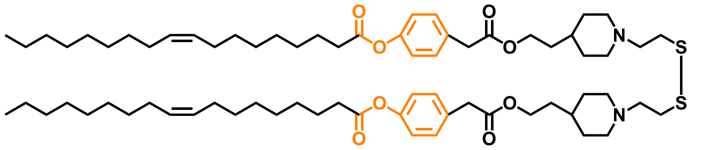

ssPalmO-Phe: reduction responsive

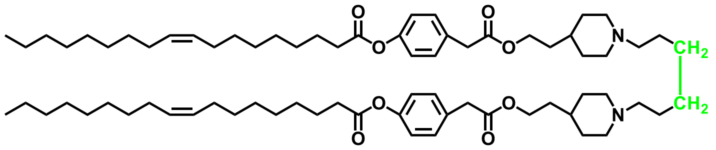

ccPalmO-Phe: reduction non-responsive

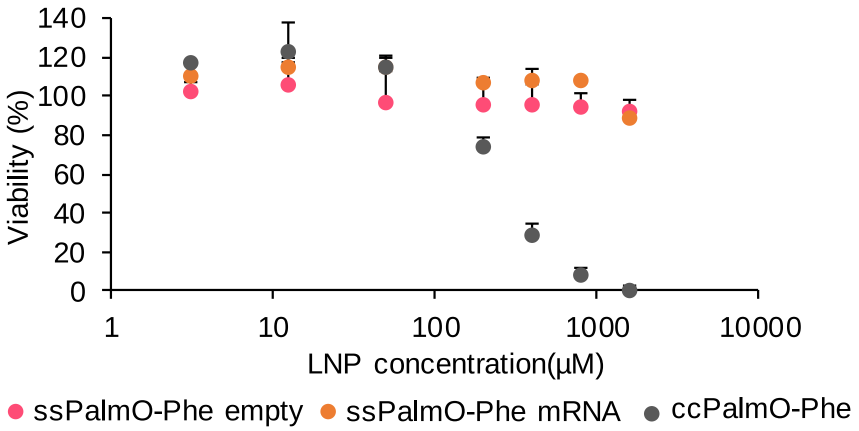

Figure S1. *In vitro* toxicity evaluation of the LNP_{ssPalm}.

Consistent with our previous results [1], the ccPalmO-Phe showed a severe toxicity against Jurkat cells at concentrations above 100 μM. In contrast, the ssPalmO-Phe-P4C2 showed no toxicity in the tested range. The maximum concentration of the lipids tested in this study (1600 M) was 8-fold higher than usual transfection condition. Based on this result, we conclude that the LNP_{ssPalm} is biocompatible with Jurkat cells.

The heterogeneity of transfection was evaluated by flow cytometry. An EGFP IVT-mRNA (TriLink Biotechnologies) was introduced to the Jurkat cells by LNP_{ssPalm} or electroporation (EP) as described in the main text. At 24h hour after transfection, the cells were collected, the cell suspension was centrifuged (500 × g, 4 °C, 3 min) and the supernatant was discarded. The cells were washed with FACS buffer (0.5 % BSA, 0.1% NaN3 in PBS) twice. The fluorescence of the EGFP was evaluated by means of a Novocyte™ flow cytometer.

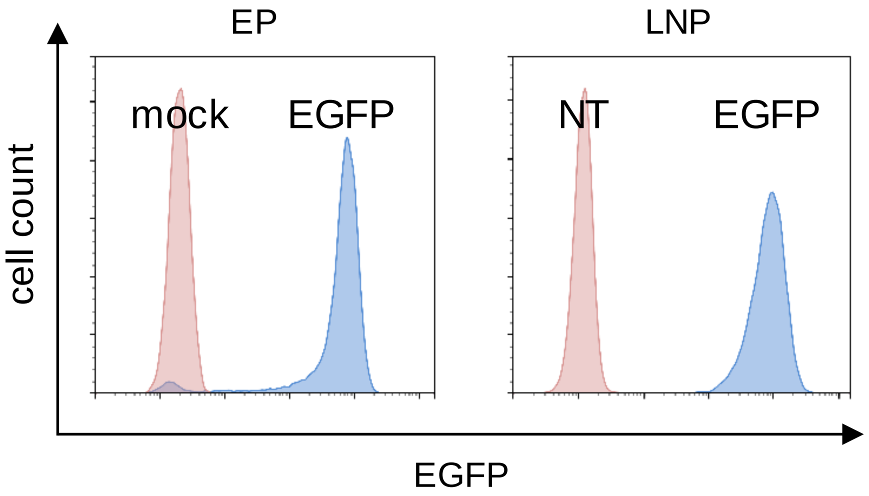

Figure S2. Homogeneous expression of mRNA by LNP_{ssPalm} and EP.

Compared to the mock-treated or non-treated cells, both electroporation and LNP showed high and homogenous expression of the EGFP.

The hemolysis activity of the LNP_{ssPalm} was evaluated as an index of the efficiency of endosomal membrane-destabilization activity. LNPs with different densities of DMG-PEG2000 (0.75%-3%) were prepared as described in the main text. The hemolytic activity was measured at pH 7.4 (physiological condition), pH 6.5 (early endosome condition), and pH5.5 (late endosome condition). The concentration of the total lipid was in the range from 12.5 μM to 200 μM. As a positive control, red blood cells were lysed by 0.1% (w/v) Triton-X100.

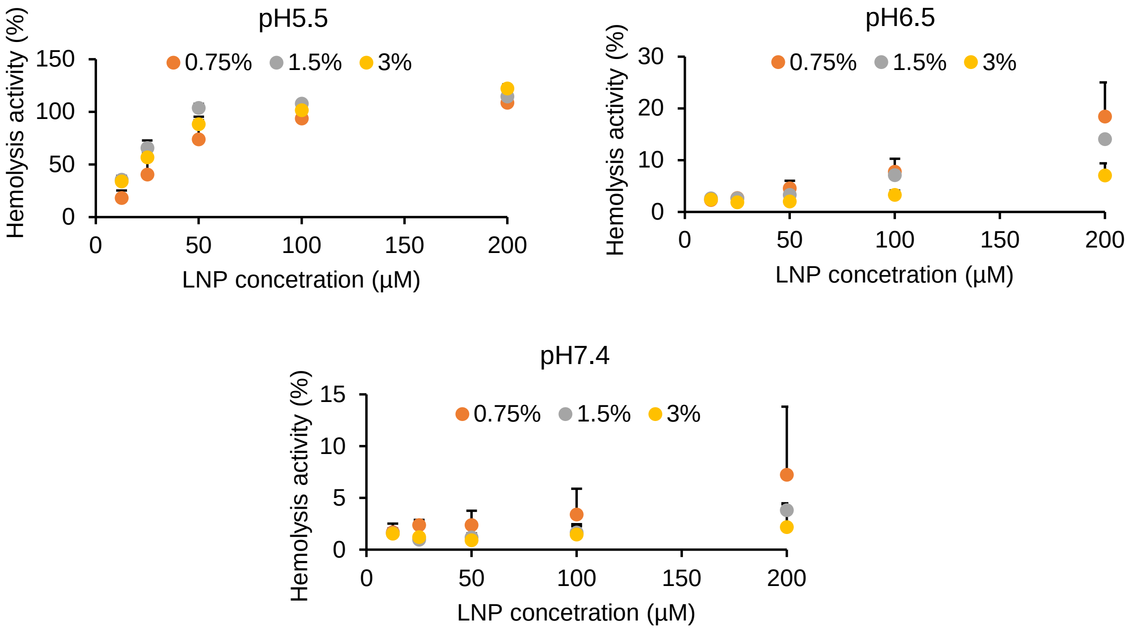

Figure S3. Effects of PEG-lipid amount on hemolysis activity.

At pH5.5 which corresponds to the late endosomal pH, all of the LNPs showed hemolysis activity and this activity was concentration dependent. At a concentration above 100 μM, the hemolysis activity reached 100%. In contrast, at pH 6.5 which corresponds to the pH of early endosomes, there was a tendency for a low amount of PEG-lipid to show a higher hemolysis activity. A similar tendency was observed at pH7.4, while the overall hemolysis activity was low. It therefore can be hypothesized that the decrease in the PEG-lipid content enhances the membrane disrupting ability of the LNPs.

The apparent pKa of the LNPs with different amounts of DMG-PEG2000 was evaluated by 6-(p-Toluidino)-2-naphthalenesulfonic acid (TNS) assay. Buffers with different pH values were prepared; 20 mM citric acid/NaOH buffer (with 150 mM NaCl, pH 3.0, 3.5, 4.0, 4.5, 5.0, 5.5), 20 mM sodium dihydrogen phosphate/NaOH buffer (with 150 mM NaCl, pH 6.0, 6.4, 6.8, 7.2, 7.6, 8.0), and 20 mM Tris/HCl buffer (with 150 mM NaCl pH8.5, 9.0, 9.5, 10.0). TNS was dissolved at 0.6 mM in water as a stock solution. In the well of 96 well-black plate, 2 μL of the TNS solution, 12 μL of the LNP solution (0.5 mM total lipid), and 186 μL of the each of the buffers above were mixed. After shaking (400 rpm, 10 min) the fluorescence of the TNS (Ex:321/Em:447) was measured. The apparent pKa of the surface was calculated as the pH at which the LNP showed 50% of the maximum fluorescence.

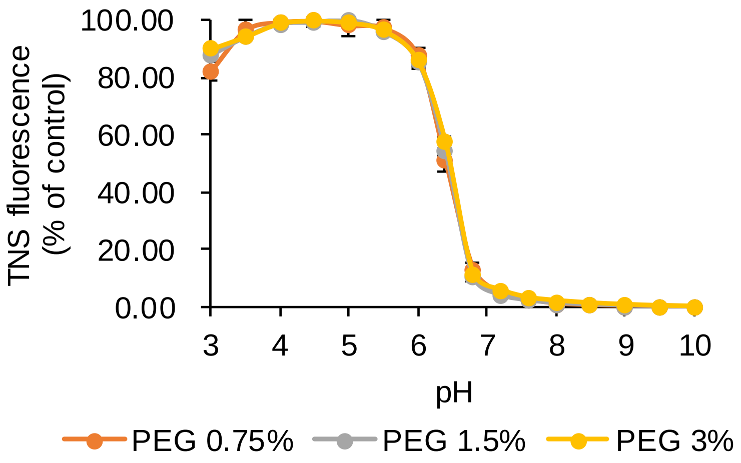

Figure S4. Apparent pKa of the LNPs with different amount of PEG-lipid.

The apparent pKa of the LNPs with 0.75% DMG-PEG2000, 1.5% DMG-PEG2000, and 3% DMG-PEG2000 were compatible; 6.41, 6.43, and 6.46, respectively. This result indicates that the development of a charge on the surface of the LNPs was not affected by the amount of PEG used. It is also indicated that the difference in the hemolysis activity shown in Figure 5 may reflect the facilitated physical interaction of the LNPs and red blood cells.

The hemolysis activity of the LNP_{ssPalm} containing POPC or POPE was compared. The hemolysis assay was conducted as described in the main text and Figure S3. The final concentration of the total lipid was 100 μM.

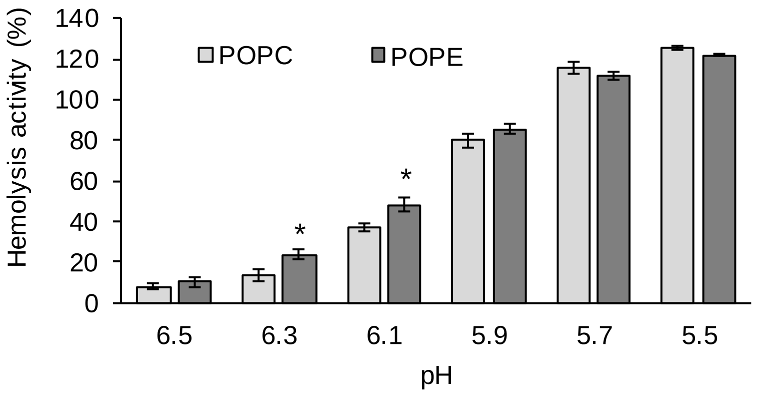

Figure S5. Effects of phospholipids on hemolysis activity.

Compared to the LNPs with POPC, LNPs with POPE showed higher hemolysis activity at pH6.3 and 6.1. Thus, the difference in the head groups also affected the endosomal escape at an early stage of endocytosis. Taken together, we conclude that the LNP_{ssPalm} with the composition of ssPalmO-Phe-P4C2/POPE/cholesterol/DMG-PEG = 50/15/35/0.75 enhanced the endosomal escape at an early stage of endocytosis.

The transfection activity of the LNP_{ssPalm} containing DOPC or POPE was compared. Raw267.4 mouse macrophage/monocyte cells and HeLa human cervical cancer cells were used for this purpose. RPMI1640 medium supplemented with 10% (*v*/*v*) FBS, 100 U/mL of penicillin, and 100 mg/mL of streptomycin. HeLa cells were cultured in D-MEM medium supplemented with 10% (*v*/*v*) FBS, 100 U/mL of penicillin, and 100 mg/mL of streptomycin. Cells were cultured in 10 cm dishes (Asnol Sterilization Petri Dish φ90 × 15mm) and were passed when they reached 80% confluence. Typical passage timing was at 2 days intervals for Raw264.7 cells and HeLa cells, respectively. The cells were cultured under an atmosphere of 5 % CO_2/air at 37 °C. The cells were incubated in culture media on the 96 well plates. In all samples, the final concentration of the mRNA and the cells were 0.0002 μg/μL and 1×10^5 cells/mL. At 24 hours after transfection, 100 μL of Steady-Glo® reagent (Promega) was added to each well. After shaking for 5 min, luminescence from 70 μL samples of the lysed samples was measured by GloMax® 20/20 Luminometer (Promega).

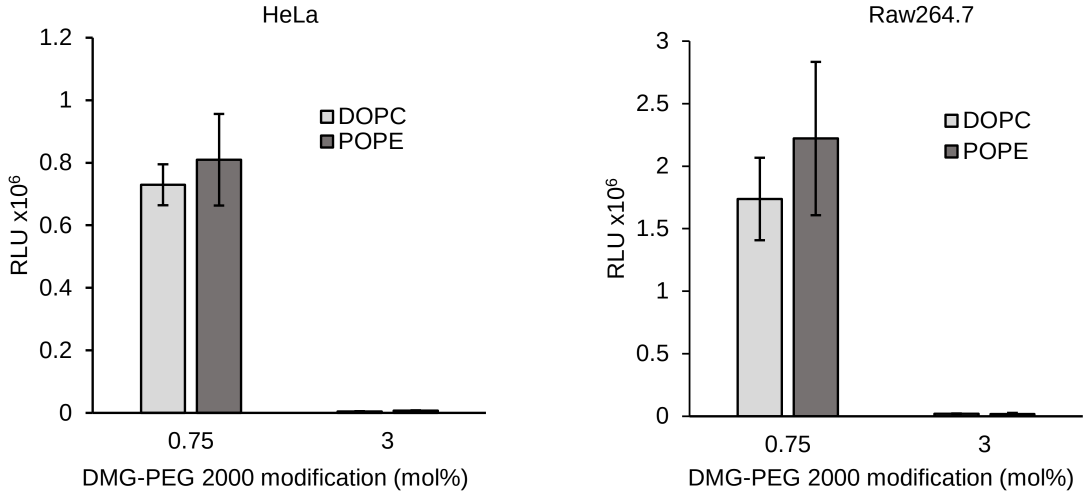

Figure S6. Transfection activity against other cell lines.

Transfection activity against HeLa Cells and Raw264.7 cells was improved by decreasing the amount of PEG-lipid. In the case of LNP_{ssPalm} containing POPE, a 115-fold and a 123-fold increase in the transfection activity for HeLa cells and Raw264.7 cells, respectively, was found. On the other hand, the difference of the DOPC and POPE was marginal for both cell lines. It can thus be concluded that the requirement of phosphatidyl ethanolamine is specific for Jurkat cells.

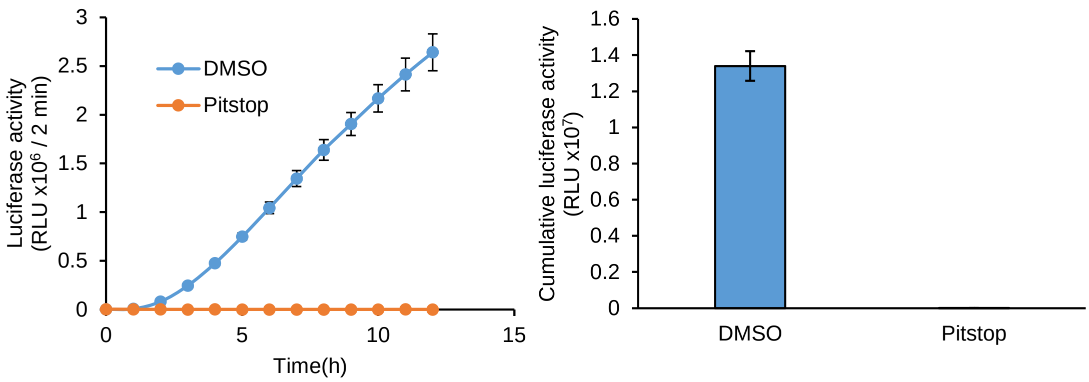

Figure S7. Transfection activity in the presence of the CME inhibitor.

The transfection activity of the LNP_{ssPalm} containing POPE was evaluated in the presence or absence of the inhibitor of clathrin-mediated endocytosis (CME). For the pre-treatment of the cells, the cells were seeded at 1 × 10^5 cells/mL in Opti-MEM. The cells were incubated with 30 μM Pitstop for 20 min. After the incubation, the cell suspension was centrifuged (4 °C, 500 × g, 5 min) and 1 mL of culture medium containing the same concentration of inhibitors was added to the pelleted cells. Cells were transfected with the LNPs containing mRNA at a concentration of 0.0002 μg/μL. The cells were placed in an incubator-type luminometer (Kronos, ATTO) and the luciferase activity was measured for 2 min at 1 h intervals.

The transfection activity of the LNP_{ssPalm} in the presence of the inhibitor was reduced nearly completely. These data indicate that cellular uptake via CME is actually important for the successful cytoplasmic delivery of mRNA.

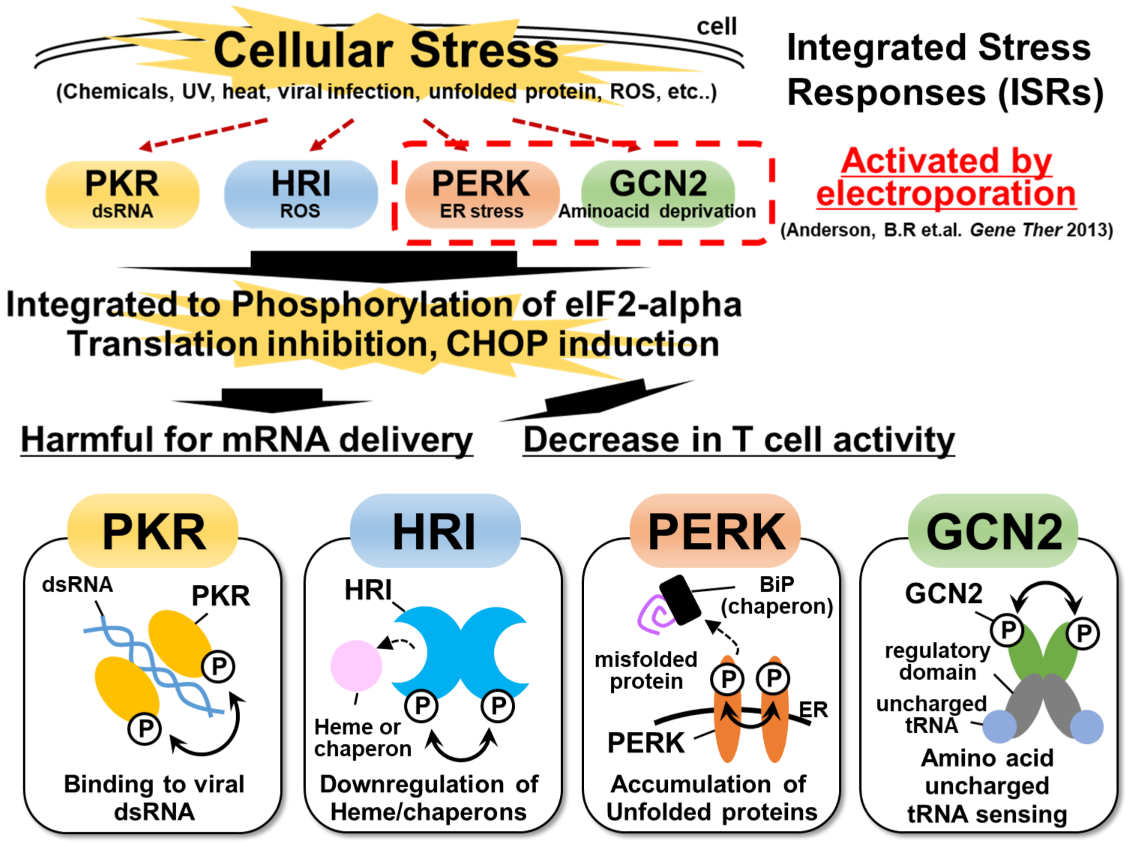

Figure S8. Schematic illustration of integrated stress responses (ISRs).

Integrated Stress Responses (ISRs) are adaptive responses of cells against diverse stresses. Stimuli such as chemicals, ultra violet right (UV), heat shock, viral infection, accumulated unfolded protein, and reactive oxygen species activated kinases called EIF2AKs including protein kinase RNA-activated (PKR), heme-regulated inhibitor (HRI), protein kinase RNA-like endoplasmic reticulum kinase (PERK), and general control non-derepressible 2 (GCN2) [2]. The representative stimuli for each EIF2AK and schematic illustration of their mechanisms of activation were shown in this figure. Although there are well-known stresses corresponding to each EIF2AKs, the specificity of the recognition of stresses has not been completely clarified. Thus, the activating stimuli for each EIF2AK are not limited to those shown above. Since the EIF2AKs share the α-subunit of eukaryotic initiation factor 2 (eIF2α) as a common target, the diverse stresses are "integrated" as the emergence of phosphorylated eIF2α (eIF2α-P). Since the eIF2α-P strongly slows down the global translation process by interacting with eukaryotic initiation factor 2B (eIF2B) [3], mRNA delivery systems have to avoid the activation of this pathway. It is well known that the incorporation of chemically modified nucleotide can avoid the recognition of extracellular mRNA by the PKR [4]. In the case of T cells, the expression of a stress related-transcription factor C/EBP homologous protein (CHOP), a consequence of PERK-dependent and tumor-induced ISRs, results in a decreased cytotoxicity of T cells [5]. Thus, the avoidance of ISRs is highly important for the successful preparation of T cell therapeutics such as CAR-T cells.

It was previously reported that electroporation induced integrated-stress-responses (ISRs) [6]. In the main text, we also showed the activation of the ISRs by the electroporation (Figure 4). In the case of electroporation, the ISRs were induced by GCN2 and PERK. GCN2 is a kinase which senses an increase in the levels of amino acid-uncharged t-RNA. The uncharged t-RNA increases in response to the decrease in available nutrients under the conditions such as serum depletion [7,8]. PERK is a kinase that senses the unfolded protein in the endoplasmic reticulum [9]. The activation of GCN2 and PERK by electroporation may indicate that the pore formation on these biological membranes results in the leakage of the contents of cytoplasm/endoplasmic reticulum into the extracellular

space. This leakage would be expected to result in the disruption in the homeostasis of cells. Compared to the EP, which caused a significant increase in the phosphorylation of the eIF2α, no elevation was observed for the transfection with the LNP_{ssPalm}. These observations indicate that the LNP_{ssPalm} with a composition of ssPalmO-Phe-P4C2/POPE/Chol/DMG-PEG02000 = 50/15/35/0.75 is a safe and effective alternative to EP.

References

1. Tanaka, H.; Takahashi, T.; Konishi, M.; Takata, N.; Gomi, M.; Shirane, D.; Miyama, R.; Hagiwara, S.; Yamasaki, Y.; Sakurai, Y.; et al. Self-Degradable Lipid-Like Materials Based on "Hydrolysis accelerated by the intra-Particle Enrichment of Reactant (HyPER)" for Messenger RNA Delivery. *Adv. Funct. Mater.* **2020**, *30*, 1910575, doi:10.1002/adfm.201910575.
2. Pakos-Zebrucka, K.; Koryga, I.; Mnich, K.; Ljujic, M.; Samali, A.; Gorman, A.M. The integrated stress response. *EMBO reports* **2016**, *17*, 1374–1395, doi:10.15252/embr.201642195.
3. Adomavicius, T.; Guaita, M.; Zhou, Y.; Jennings, M.D.; Latif, Z.; Roseman, A.M.; Pavitt, G.D. The structural basis of translational control by eIF2 phosphorylation. *Nature communications* **2019**, *10*, 2136, doi:10.1038/s41467-019-10167-3.
4. Anderson, B.R.; Muramatsu, H.; Nallagatla, S.R.; Bevilacqua, P.C.; Sansing, L.H.; Weissman, D.; Kariko, K. Incorporation of pseudouridine into mRNA enhances translation by diminishing PKR activation. *Nucleic Acids Res.* **2010**, *38*, 5884–5892, doi:10.1093/nar/gkq347.
5. Cao, Y.; Trillo-Tinoco, J.; Sierra, R.A.; Anadon, C.; Dai, W.; Mohamed, E.; Cen, L.; Costich, T.L.; Magliocco, A.; Marchion, D.; et al. ER stress-induced mediator C/EBP homologous protein thwarts effector T cell activity in tumors through T-bet repression. *Nature communications* **2019**, *10*, 1280, doi:10.1038/s41467-019-09263-1.
6. Anderson, B.R.; Kariko, K.; Weissman, D. Nucleofection induces transient eIF2alpha phosphorylation by GCN2 and PERK. *Gene Ther.* **2013**, *20*, 136–142, doi:10.1038/gt.2012.5.
7. Castilho, B.A.; Shanmugam, R.; Silva, R.C.; Ramesh, R.; Himme, B.M.; Sattlegger, E. Keeping the eIF2 alpha kinase Gcn2 in check. *Biochim. Biophys. Acta* **2014**, *1843*, 1948–1968, doi:10.1016/j.bbamcr.2014.04.006.
8. Anda, S.; Zach, R.; Grallert, B. Activation of Gcn2 in response to different stresses. *PLoS ONE* **2017**, *12*, e0182143, doi:10.1371/journal.pone.0182143.
9. Gonen, N.; Sabath, N.; Burge, C.B.; Shalgi, R. Widespread PERK-dependent repression of ER targets in response to ER stress. *Scientific reports* **2019**, *9*, 4330, doi:10.1038/s41598-019-38705-5.
